# Modeling QTL-by-environment interactions for multi-parent populations

**DOI:** 10.3389/fpls.2024.1410851

**Published:** 2024-07-31

**Authors:** Wenhao Li, Martin P. Boer, Ronny V. L. Joosen, Chaozhi Zheng, Lawrence Percival-Alwyn, James Cockram, Fred A. Van Eeuwijk

**Affiliations:** ^1^ Biometris, Wageningen University and Research Center, Wageningen, Netherlands; ^2^ Rijk Zwaan Breeding B.V., De Lier, Netherlands; ^3^ Plant Genetics, NIAB, Cambridge, United Kingdom

**Keywords:** multi-parent population, diallel, NAM, MAGIC, QTL-by-environment interaction, multienvironment trial, maize, wheat

## Abstract

Multi-parent populations (MPPs) are attractive for genetic and breeding studies because they combine genetic diversity with an easy-to-control population structure. Most methods for mapping QTLs in MPPs focus on the detection of QTLs in single environments. Little attention has been given to mapping QTLs in multienvironment trials (METs) and to detecting and modeling QTL-by-environment interactions (QEIs). We present mixed model approaches for the detection and modeling of consistent versus environment-dependent QTLs, i.e., QTL-by-environment interaction (QEI). QTL effects are assumed to be normally distributed with variances expressing consistency or dependence on environments and families. The entries of the corresponding design matrices are functions of identity-by-descent (IBD) probabilities between parents and offspring and follow from the parental origin of offspring DNA. A polygenic effect is added to the models to account for background genetic variation. We illustrate the wide applicability of our method by analyzing several public MPP datasets with observations from METs. The examples include diallel, nested association mapping (NAM), and multi-parent advanced inter-cross (MAGIC) populations. The results of our approach compare favorably with those of previous studies that used tailored methods.

## 1 Introduction

Genotype-by-environment interaction (GEI) implies the differential behavior of genotypes across a range of environmental conditions. Broadly adapted genotypes show stable performance across environmental conditions, whereas narrowly adapted genotypes do well under specific conditions. Adequate description and understanding of GEI patterns is fundamental to the creation of better adapted varieties that comply with environmental and societal challenges. Obviously, adaptation to conditions imposed by climate change will be a major target for many breeding programs.

GEI is a common phenomenon in many crops, and cereals are no exception to that. Typically, a diversity panel is evaluated in a multi-environment trial (MET) or a number of managed stress trials and the GEI is explained in terms of marker or QTL effects that are environment dependent. Some recent examples of this approach using a QTL analysis in the form of a genome-wide association analysis are Bustos-Korts et al. (2019) and Bretani et al. (2022) in barley and Millet et al. (2016) and Shu et al. (2023) in maize. Examples of the same approach with an emphasis on genomic prediction are Burgueño et al. (2012); Lopez-Cruz et al. (2015), and Jarquín et al. (2017) in wheat and Millet et al. (2019) and Barreto et al. (2024) in maize, whereas Cuevas et al. (2017) give examples in wheat and maize. It is less common to use biparental populations for the investigation of the genetic bases of GEI in the form of QTL by environment interactions (QEI). Two somewhat older examples are Boer et al. (2007) for maize and Mathews et al. (2008) for wheat. All the above papers use a linear mixed model approach or a Bayesian equivalent to model the data. For a description of such models in relation to the modeling of GEI and QEI, see van Eeuwijk et al. (2010); Malosetti et al. (2013), and Van Eeuwijk et al. (2016).

The utilization of multi-parent populations (MPPs) in the investigation of QEI offers several advantages. Compared with the use of biparental populations in METs, the higher genetic diversity of MPPs will increase the chance of displaying polymorphisms at QTLs that interact with environments. In comparison with diversity panels, the known pedigree of MPPs alleviates the problems of population structure and minor allele frequencies. A few papers have attempted to analyze multi-environment trials for multi-parent populations (MET&MPP) with often QTL analyses per environment and subsequent comparison of QTL test statistics or *−log10(p-value*) profiles. An attractive variation on this single-environment single-response QTL analysis was given by Coles et al. (2010) for a maize diallel design where QTLs for the photoperiod sensitivity were mapped using an integrated response variable, i.e., the difference between observations under long-day and short-day conditions was analyzed (Coles et al., 2010). In a maize NAM design, QTL effects for yield were assessed between two weakly correlated trial locations with consideration of the genetic covariance between environments (Garin et al., 2020). Other examples of maize NAM and diallel MET&MPPs (Buckler et al., 2009; Giraud et al., 2014) demonstrated QTL detection for the average response across environments, ignoring QEI. For MAGIC populations, Verbyla et al. (2014) present mixed model technology to study QEI. Puglisi et al. (2021) analyzed a barley MAGIC population in a MET for genomic prediction.

A generic statistical approach for studying QEI for any type of MPP seems to be missing. We propose such an approach in the form of an IBD-based mixed model framework with random QTL effects whose stability depends on both the environment and the family. A background polygenic effect is added that itself can be structured again by family and environment. We illustrate our approach in several MET&MPP cereal datasets: a maize diallel (Coles et al., 2010), two maize NAM designs (Bauer et al., 2013; Giraud et al., 2014; Garin et al., 2020), and a wheat MAGIC population (Scott et al., 2021).

## 2 Materials and methods

### 2.1 General framework

**Figure 1:**
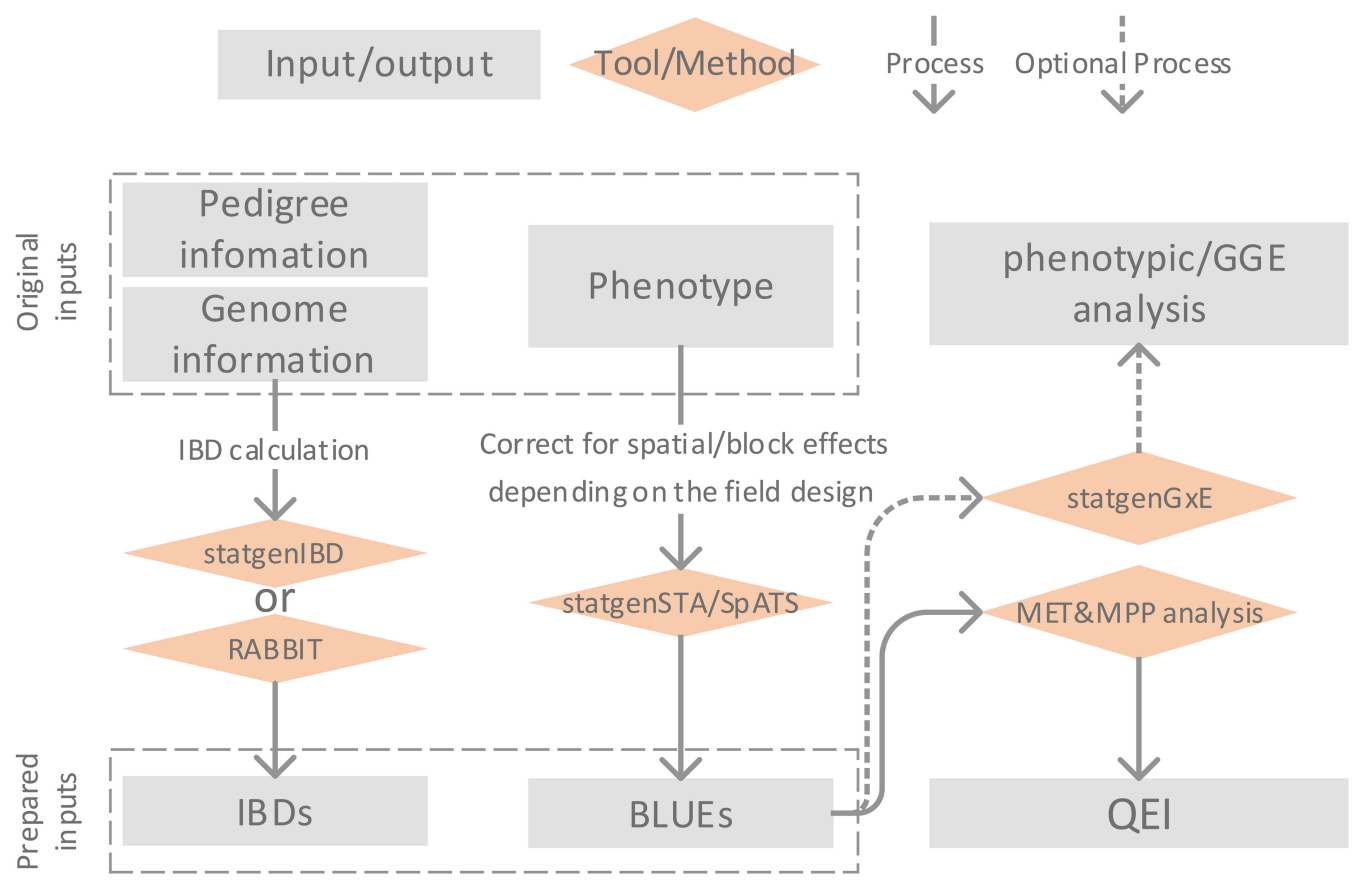
Description of framework for Identity By Descent (IBD)-based QTL analysis of Multi-Environment Trial (MET) data collected for a Multi-Parent Population (MPP) allowing the study of QTL by environment interactions (QEI).

**Figure 1** illustrates the framework of MET&MPP analysis for investigating QEI. The process begins with the computation of IBD probabilities using pedigree and genome information of parents and progenies. Next, the best linear unbiased estimates (BLUEs) for genotypic means are obtained through single-trial analysis, which includes corrections for block and spatial effects. Subsequently, a MET&MPP analysis is conducted using mixed model approaches to study QEI. An important issue in the building of mixed models for GEI and QEI in METs is the formulation of the variance–covariance (VCOV) structure between trials (Smith et al., 2005; Boer et al., 2007). An informal exploration of this structure is possible via the fitting of a genotype-plus-genotype-by-environment interaction (GGE) model and the corresponding visualization by a GGE biplot (Yan et al., 2000; Yan and Kang, 2002). Details of each step in **Figure 1** are described below, and descriptions of the proposed IBD-based MET&MPP analysis using mixed models for QEI are elaborated in section 2.2 *“*Mixed models for IBD-based QTL analysis of METs for MPPs*”*.

The entries of design matrices, representing genetic predictors for testing QTL main effects and QEI, are derived from marker information and have the form of expected numbers of alleles originating from individual parents. These expected allele numbers are simple functions of IBD probabilities between parents and offspring. IBD probabilities can be calculated using the R package *statgenIBD* (Boer and van Rossum, 2021) for a wide range of MPP designs, whereas a Mathematica tool called RABBIT can be used for more complex designs (Zheng, 2019). Both *RABBIT* and *statgenIBD* employ hidden Markov models (HMM) and inheritance vectors to calculate IBD probabilities based on the pedigree and genome information of parents and offspring (Zheng et al., 2014, 2015; Boer and van Rossum, 2021). In the current paper, we chose to compute IBD probabilities at a grid of 5 cM along the genome for the empirical MET&MPP datasets as a compromise between mapping resolution and computation time. The exception was the analysis of a MAGIC wheat data set for which we defined a grid at 0.5 cM.

The multi-environment phenotypes used as inputs to our MET&MPP models are typically genotypic means for offspring belonging to segregating families evaluated in multiple field trials. Trial-specific genotypic means, which have been corrected for block, row, column, and spatial effects, are calculated as best linear unbiased estimators (BLUEs). The R package *SpATS* (Rodriguez-Alvarez et al., 2018) offers a convenient and efficient way to convert the trial data at plot level into vectors of adjusted genotypic means. Similarly, the R package *statgenSTA* (Rossum et al., 2021b) can be employed to compute genotypic BLUEs per trial together with corresponding standard errors.

To explore the heterogeneity of genotypic variances and correlations across trials, we performed a GGE biplot analysis (Yan et al., 2000). The GGE biplot is a rank 2 principal component fit to the genotype-by-trial table of BLUEs in which the first axis is closely related to the genotypic main effects whereas the second axis shows GEI effects. The R package *statgenGxE* (Rossum et al., 2021a) was used to create the GGE biplots. These plots can be helpful to investigate whether the contributions of individual QTLs to the genetic correlations between trials align with the overall correlations.

### 2.2 Mixed models for IBD-based QTL analysis of METs for MPPs

#### 2.2.1 Models for QTL effects

Four QTL models are proposed differing in the effect types at a putative QTL in terms of stability across families and environments: consistent across both environments and families (environment-consistent and family-consistent, EC&FC QTL), environment-specific and family-consistent (ES&FC QTL), environment-consistent and family-specific (EC&FS QTL), or environment-specific and family-specific (ES&FS QTL). For MAGIC populations, we consider the consistency and specificity of QTL effects only in relation to the environments.

We elaborate these four QTL models for a MET&MPP with *C* crosses (or families) derived from *P* parents across *J* environments or trials; $n_{cj}$ denotes the number of observations (genotypic BLUEs) for the *c*-th family in the *j*-th environment; the total number of observations from the *c*-th family across all *J* environments is $\sum_{j}n_{cj}=n_{c\cdot }$ ; the total number of observations in the *j*-th environment across all *C* families is $\sum_{c}n_{cj}=n_{\cdot j}$ ; so the total number of observations in the MET&MPP is $\sum_{j}\sum_{c}n_{cj}=\sum_{c}n_{c\cdot }=\sum_{j}n_{\cdot j}=N$ . In all model descriptions, we present later, matrices (and vectors) are presented in bold font and random terms are underlined. The general mixed model for a single-locus QTL model at the *q*-th genomic position can be expressed as:


$$\underline{\text{Y}}=\text{X}\beta +{\text{Z}}_{\text{q}}{\underline{\text{u}}}_{q}+\underline{\text{g}}+\underline{\text{ϵ}}$$

$\underline{\text{Y}}$ is the N×1 column vector for all *N* observations in a MET&MPP. The fixed part, Xβ , models effects for families and environments and their interactions. The structure of the design matrix for the random QTL effects, ${\text{Z}}_{\text{q}}$ , and the vector of QTL effects, ${\underline{\text{u}}}_{q}$ , is determined by the effect type at the QTL. The four QTL models have different design matrices ${\text{Z}}_{q}$ and QTL effect vectors ${\underline{\text{u}}}_{q}$ and will be described with superscripts to indicate the matrix dimensions to help distinguish the model structures.

In the EC&FC QTL model, the QTL effect at the *q*-th genomic position is assumed to be stable across environments and families, which is comparable with the generic IBD-based mixed model approach to map additive QTLs in previous studies (Li et al., 2021, 2022):


$${\text{Z}}_{q}^{\left(N\times P\right)}=\left[{\text{π}}_{q,1}^{\left(N\times 1\right)}\ldots {\text{π}}_{q,k}^{\left(N\times 1\right)}\ldots {\text{π}}_{q,P}^{\left(N\times 1\right)}\right],$$

$${\underline{\text{u}}}_{q}^{\left(P\times 1\right)}={\left[{\underline{a}}_{q,1}\ldots {\underline{a}}_{q,k}\ldots {\underline{a}}_{q,P}\right]}^{T}∼\;MVN\left(0,\;{\text{I}}_{P}{\text{Σ}}_{q}^{2}\right)$$

(EC&FC QTL model)

${\text{Z}}_{q}^{\left(N\times P\right)}$ is a N×P design matrix that can be partitioned into *P* column vectors. Each of the *P* column vectors is a N×1 column vector, ${\text{π}}_{q,k}^{\left(N\times 1\right)}$ , containing expected numbers of allelic copies associated with the *k*-th ( k=1,2,…, P ) parent for all *N* observations. The P×1 column vector ${\underline{\text{u}}}_{q}^{\left(P\times 1\right)}$ contains element ${\underline{a}}_{q,k}$ representing the QTL effect associated with the *k*-th parent. The variance of ${\underline{\text{u}}}_{q}^{\left(P\times 1\right)}$ is ${\text{I}}_{P}{\text{Σ}}_{q}^{2}$ , where ${\text{I}}_{P}$ is the *P*-dimensional identity matrix and ${\text{Σ}}_{q}^{2}$ is the genetic variance for the putative QTL, implying a homogeneous VCOV structure for the QTL effect across all environments and families.

In the ES&FC QTL model, the QTL effect is defined as being unstable across *J* environments due to QEI but consistent across *C* families. The design matrix and the QTL effects become:


$${\text{Z}}_{q}^{\left(N\times PJ\right)}={⊕}_{j=1}^{J}{\text{Z}}_{q,j}^{\left(n_{\cdot j}\times P\right)}$$

$${\underline{u}}_{q}^{\left(PJ\times 1\right)}={\left[{\left({\underline{u}}_{q,1}^{\left(P\times 1\right)}\right)}^{T}\ldots {\left({\underline{u}}_{q,j}^{\left(P\times 1\right)}\right)}^{T}\ldots {\left({\underline{u}}_{q,J}^{\left(P\times 1\right)}\right)}^{T}\right]}^{T}∼MVN\left(0,{⊕}_{j=1}^{J}I_{P}{\text{Σ}}_{q,j}^{2}\right)$$

(ES&FC QTL model)

${\text{Z}}_{q}^{\left(N\times PJ\right)}$ is a N×PJ design matrix that can be split into *J* diagonal components. Each of these *J* components is a $n_{\cdot j}\times P$ design matrix, ${\text{Z}}_{q,j}^{\left(n_{\cdot j}\times P\right)}$ , whose entries correspond to an $n_{\cdot j}\times P$ sub matrix of ${\text{Z}}_{q}^{\left(N\times P\right)}$ , i.e., the entries corresponding to the *j*-th environment in ${\text{Z}}_{q}^{\left(N\times P\right)}$ are copied to ${\text{Z}}_{q,j}^{\left(n_{\cdot j}\times P\right)}$ , with $n_{\cdot j}$ being the number of observations in the *j*-th environment. The vector of effects ${\underline{\text{u}}}_{q}^{\left(PJ\times 1\right)}$ is a PJ×1 column vector that can be partitioned into *J* sub vectors. Each of the *J* sub vectors is a P×1 column vector ${\underline{\text{u}}}_{q,j}^{\left(P\times 1\right)}$ that contains the QTL effects of the *P* parents in the *j*-th environment with ${\text{I}}_{P}{\text{Σ}}_{q,j}^{2}$ being the environment-specific QTL variance. The ⊕ symbol stands for a direct sum (Schott, 2016).

In the EC&FS QTL model, parental QTL effects are specified within each of *C* biparental families and therefore vary between families, while being stable across all *J* environments. The design matrix for the QTL effects and the vector of QTL effects are defined as follows:


$$Z_{q}^{\left(N\times 2C\right)}={⊕}_{c=1}^{C}\left[\pi_{q,P1_{c}}^{\left(n_{c\cdot }\times 1\right)}\;\pi_{q,P2_{c}}^{\left(n_{c\cdot }\times 1\right)}\right]$$

$${\underline{u}}_{q}^{\left(2C\times 1\right)}={\left[{\underline{a}}_{q,P1_{1}}\;{\underline{a}}_{q,P2_{1}}\ldots {\underline{a}}_{q,P1_{c}}\;{\underline{a}}_{q,P2_{c}}\ldots {\underline{a}}_{q,P1_{C}}\;{\underline{a}}_{q,P2_{C}}\right]}^{T}\sim MVN\left(0,{⊕}_{c=1}^{C}I_{2}{\text{Σ}}_{q,c}^{2}\right)$$

(EC&FS QTL model)

${\text{Z}}_{q}^{\left(N\times 2C\right)}$ is a N×2C design matrix that can be partitioned into *C* diagonal components. Each of these components contains two $n_{c.}\times 1$ column vectors, ${\text{π}}_{q,P1_{c}}^{\left(n_{c\cdot }\times 1\right)}$ and ${\text{π}}_{q,P2_{c}}^{\left(n_{c\cdot }\times 1\right)}$ , whose elements indicate the expected numbers of allele copies associated with the two parents of that family, $P1_{c}$ and $P2_{c}$ . The random QTL effects ${\underline{\text{u}}}_{q}^{\left(2C\times 1\right)}$ form a 2C×1 column vector associated with the pairs of parents across the families, and for the *c*-th family, the variance of the QTL effects is ${\text{I}}_{2}{\text{Σ}}_{q,c}^{2}$ .

In the ES&FS QTL model, the QTL effects are stable across neither environments nor families, but both are environment and family specific. The ES&FS QTL model is established by merging the ES&FC and EC&FS QTL models. The design matrix for the QTL effects and the corresponding vector of QTL effects is:


$$Z_{q}^{\left(N\times 2CJ\right)}={⊕}_{j=1}^{J}{⊕}_{c=1}^{C}\left[\pi_{q,P1_{c}}^{\left(n_{cj}\times 1\right)}\;\pi_{q,P2_{c}}^{\left(n_{cj}\times 1\right)}\right],$$

$${\underline{u}}_{q}^{\left(2JC\times 1\right)}={\left[{\left({\underline{u}}_{q,1}^{\left(2C\times 1\right)}\right)}^{T}\ldots {\left({\underline{u}}_{q,j}^{\left(2C\times 1\right)}\right)}^{T}\ldots {\left({\underline{u}}_{q,J}^{\left(2C\times 1\right)}\right)}^{T}\right]}^{T}∼MVN\left(0,{⊕}_{j=1}^{J}{⊕}_{c=1}^{C}I_{2}{\text{Σ}}_{q,j,c}^{2}\right)$$

(ES&FS QTL model)

${\text{Z}}_{q}^{\left(N\times 2CJ\right)}$ is a N×2CJ design matrix with *JC* diagonal components. Each of these components possesses a pair of $n_{cj}\times 1$ column vectors, ${\text{π}}_{q,P1_{c}}^{\left(n_{cj}\times 1\right)}$ and ${\text{π}}_{q,P2_{c}}^{\left(n_{cj}\times 1\right)}$ , whose structure resembles that in the EC&FS QTL model, but now the vectors are designated for the *j*-th environment, where $n_{cj}$ is the number of observations in the *c*-th family and *j*-th environment. Remember that $\sum_{j=1}^{J}\sum_{c=1}^{C}n_{cj}=N$ . The 2JC×1 column vector ${\underline{\text{u}}}_{q}^{\left(2JC\times 1\right)}$ can be partitioned into *J* column vectors with each column vector having the form ${\underline{\text{u}}}_{q,j}^{\left(2C\times 1\right)}$ , comparable with that in the EC&FS QTL model. For the *j*-th environment, the variance of the QTL effects across the *C* families can be written as ${⊕}_{c=1}^{C}{\text{I}}_{2}{\text{Σ}}_{q,j,c}^{2}$ . QTL variances are heterogeneous and depend on environment and family simultaneously.

#### 2.2.2 Models for polygenic effect

The QTL models above can be combined in single- or multi-QTL models with a structured polygenic effect, $\underline{\text{g}}$ . We write for the VCOV of $\underline{\text{g}}$ , ${\text{Σ}}_{g}={\text{Σ}}_{MET}⊗{\text{Σ}}_{MPP}$ , with ${\text{Σ}}_{MPP}$ defined by the relations between the families and ${\text{Σ}}_{MET}$ by the relations between the environments. For ${\text{Σ}}_{MPP}$ , we allowed for identity, i.e., homogeneity across families ( ${\text{Σ}}_{MPP_{id}}$ ), heterogeneity across families ( ${\text{Σ}}_{MPP_{idh}}$ ), or a population structure as a marker-based kinship matrix ( ${\text{Σ}}_{MPP_{kin}}$ ). For ${\text{Σ}}_{MET}$ , we assumed identity or homogeneity of genetic variance across environments and no genetic correlation between environments ( ${\text{Σ}}_{MET_{id}}$ ), heterogeneity of genetic variance across environments and no genetic correlation ( ${\text{Σ}}_{MET_{idh}}$ ), and an unstructured model with environment-specific genetic variances and correlations ( ${\text{Σ}}_{MET_{us}}$ ). The combination of three structure models for ${\text{Σ}}_{MPP}$ and three structure models for ${\text{Σ}}_{MET}$ generates nine VCOV structures for the polygenic background effect. However, in practice for our data, the polygenic model that worked best in all cases was ${\text{Σ}}_{g}={\text{Σ}}_{MET_{us}}⊗{\text{Σ}}_{MPP_{idh}}$ .

#### 2.2.3 Model selection and genome-wide QTL scans

To conduct genome-wide QTL scans, several strategies can be employed. We adopted the following protocol. Based on preliminary analyses, we first chose the VCOV model for the polygenic effects in all genome-wide scans ${\text{Σ}}_{g}={\text{Σ}}_{MET_{us}}⊗{\text{Σ}}_{MPP_{idh}}$ . We then conducted four series of full genome scans, one for each type of QTL (EC&FC, ES&FC, EC&FS, and ES&FS). Within each series of scans, we performed significance tests for QTLs using likelihood ratio tests (LRT) for single-variance components. The corresponding *p-value* followed from an approximation of the test statistic by a mixture of χ^2^ distributions with 0 and 1 degrees of freedom (Self and Liang, 1987). A conservative Bonferroni-corrected significance threshold was used at a genome-wide level of 0.05. Within a series of scans for a particular QTL type, in the first round, we selected the most significant QTL with the highest *−log10(p-value)* as a cofactor for a second round. In this second round, an exclusion window of 20 cM around the cofactor was defined in which no tests for QTLs were performed. This is to avoid collinearity problems. The process of identifying cofactors was repeated in subsequent rounds of scans until no further QTLs were found and the *−log10(p-value)* profile stabilized. This procedure can be thought of as a forward regression variable selection procedure. For each particular type of QTL (EC&FC, ES&FC, EC&FS, and ES&FS), a multi-QTL model was produced that comprised all of the QTLs identified in the series of scans. The QTLs as appearing in the four final multi-QTL models corresponding to the four specific QTL types were then combined within one model. When in this combined model QTLs of different types (EC&FC, ES&FC, EC&FS, and ES&FS) coincided with respect to their position, we assessed for that position the nature of the QTL by comparing AIC values (Akaike, 1974) for models with different formulations for the QTL effect. The model with the smallest AIC value then determined the nature of the QTL effect.

### 2.3 Data

To test our approach, we collected several empirical MET&MPP datasets (**Table 1**) including a wheat MAGIC population, a maize diallel, and two maize NAM designs. The wheat MAGIC population was phenotyped in two field trials in the UK in 2017 and 2018. The maize diallel designs were screened in managed stress trials, whereas the two maize NAM designs were screened across several geographic locations in the EU. In this paper, we did not distinguish the two types of trials and use the term “environments” to describe the trial conditions.

**Table 1:** Overview of MET&MPP data sets. Structure, size, traits, nature of phenotypic data, and reference to earlier data presentations and analyses.

MPPs	Size	Traits	METs	Reference
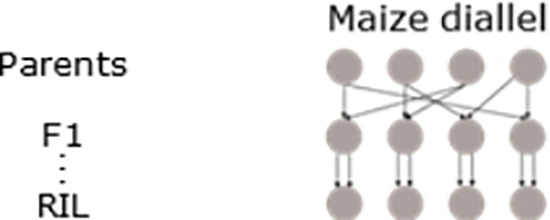	569	Growing degree days to silking (GDDTS) and anthesis (GDDTA)	Summer and winter seasons	Coles et al., 2010
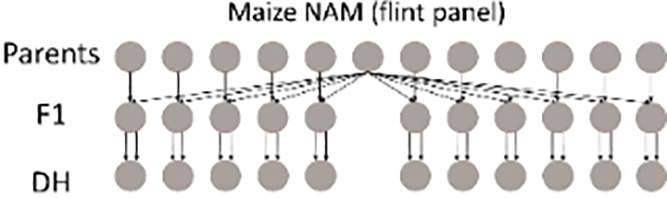	811	Dry matter yield (DMY), plant height (PH), days to silking (DtSILK)	Six geographic locations across EU	Giraud et al., 2014; Garin et al., 2020
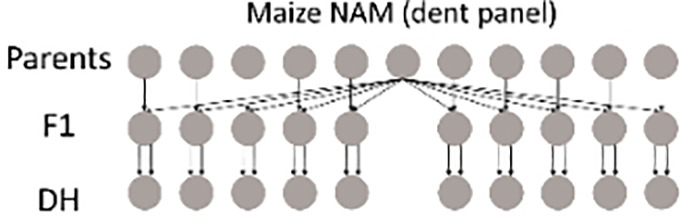	841	Dry matter yield (DMY), plant height (PH), days to silking (DtSILK)	Four geographic locations across EU	Giraud et al., 2014
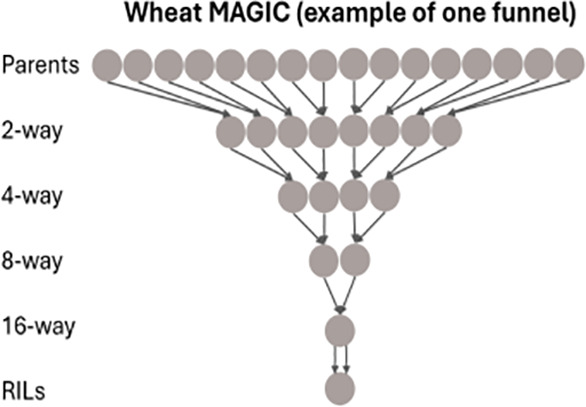	504	Grain yield (GY), grain protein content (GPC), height to flag leaf base (HFLB), flag leaf to ear distance (FLED)	Two year at same UK location. Environment 1 = 2016-2017 season. Environment 2 = 2017-2018 season	Scott et al., 2021

#### 2.3.1 Maize diallel

In the maize diallel design, four biparental families were created by crossing each of two temperate inbred parents to each of two photoperiod-sensitive tropical parents (Coles et al., 2010). Recombinant inbred lines (RILs) were screened under long-day (summer seasons) and short-day (winter seasons) conditions for photoperiod-related traits, e.g., days to silking (DTS) and anthesis (DTA), whose measurements were converted to growing degree days (GDDTS and GDDTA) to account for the influence of temperature. In the previous study (Coles et al., 2010), QTL mapping for photoperiodic responses was performed by calculating the difference in responses between summer and winter conditions. In addition, QTL mapping for the separate conditions was performed by the tool MCQTL 4.0 (Jourjon et al., 2005).

#### 2.3.2 Maize NAM, dent and flint panels

Two maize NAM designs, a flint panel and a dent panel, were taken from the maize EU-NAM project (Bauer et al., 2013; Giraud et al., 2014; Lehermeier et al., 2014). In the flint panel, 11 doubled haploid (DH) families were derived from the central parent UH007 crossed with 11 peripheral parents. Multiple traits such as dry matter yield (DMY), days to silking (DtSILK), and plant height (PH) were measured across six locations in the EU, namely, Wadersloh (Germany), Ploudaniel (France), La Coruña (Spain), Einbeck (Germany), Roggenstein (Germany), and Eckartseier (Germany). In the dent panel, 10 families were derived from the central parent F353 which was crossed with 10 peripheral parents. The same traits as measured in the flint panel were measured again but across four locations only, namely, Wadersloh (Germany), Mons (Germany), Einbeck (Germany), and Roggenstein (Germany).

In previous work by Giraud et al. (2014), combined linkage and linkage disequilibrium mapping was performed for both NAM panels, whereas Garin et al. (2020) used the NAM flint panel as an example for a QEI study using only the Roggenstein and La Coruña locations.

#### 2.3.3 Wheat MAGIC

Construction, genotyping, and phenotyping of the bread wheat (*Triticum aestivum* L.) cultivars in the NIAB Diverse MAGIC population was previously described (Scott et al., 2021). Briefly, 16 northwestern wheat cultivars were selected based on genetic diversity as founders. These were inter-crossed using a funnel design scheme across four generations, with the outputs of the crossing funnel selfed over six generations to produce 504 recombinant inbred lines (RILs). The founders were genotyped via exome capture and the RILs via whole-genome low-coverage sequencing, allowing 1.1 M high-quality single-nucleotide polymorphisms (SNPs) to be called in the RILs via imputation (Scott et al., 2021). The populations were phenotyped for agronomic traits in two field trials conducted in the United Kingdom in 2017 and 2018, as described by Scott et al. (2021). Although genome wide scans for QEI were performed for all traits recorded in both years, in this paper we will focus on QTLs close to the long-day photoperiod response locus *Photoperiod-B1* (*Ppd-B1*) on chromosome 2B for the traits grain yield (GY), grain protein content (GPC), flag leaf to ear distance (FLED), and height to flag leaf base (HFLB). Earlier QTL analyses on the same data were performed by Scott et al. (2021) for all trait-by-year combinations, where neither QEI issues nor allelic differences between parents were addressed.

## 3 Results

### 3.1 Detecting QEI from QTL models

We present the results of QEI analysis for the maize diallel, two maize NAM MET&MPPs, and for the NIAB Diverse wheat MAGIC population. For the maize diallel and NAM populations, we emphasize genome-wide QTL analyses. We also examined the GGE biplot to explore the genotypic correlations between the environments. Environments with weak correlations and therefore high GEI will reveal underlying QEI effects. We inspected environment-specific QTLs to see whether their effect profiles corresponded to the genotypic correlations observed between the environments. For the wheat MAGIC, we concentrated on the interpretation of allele effect profiles for a pleiotropic QTL on chromosome 2B, more specifically the region around the *Ppd-B1* locus. We compared our findings with previous studies that employed different methods to investigate QEI.

### 3.2 Maize diallel design for days to silking and anthesis

**Figure 2:**
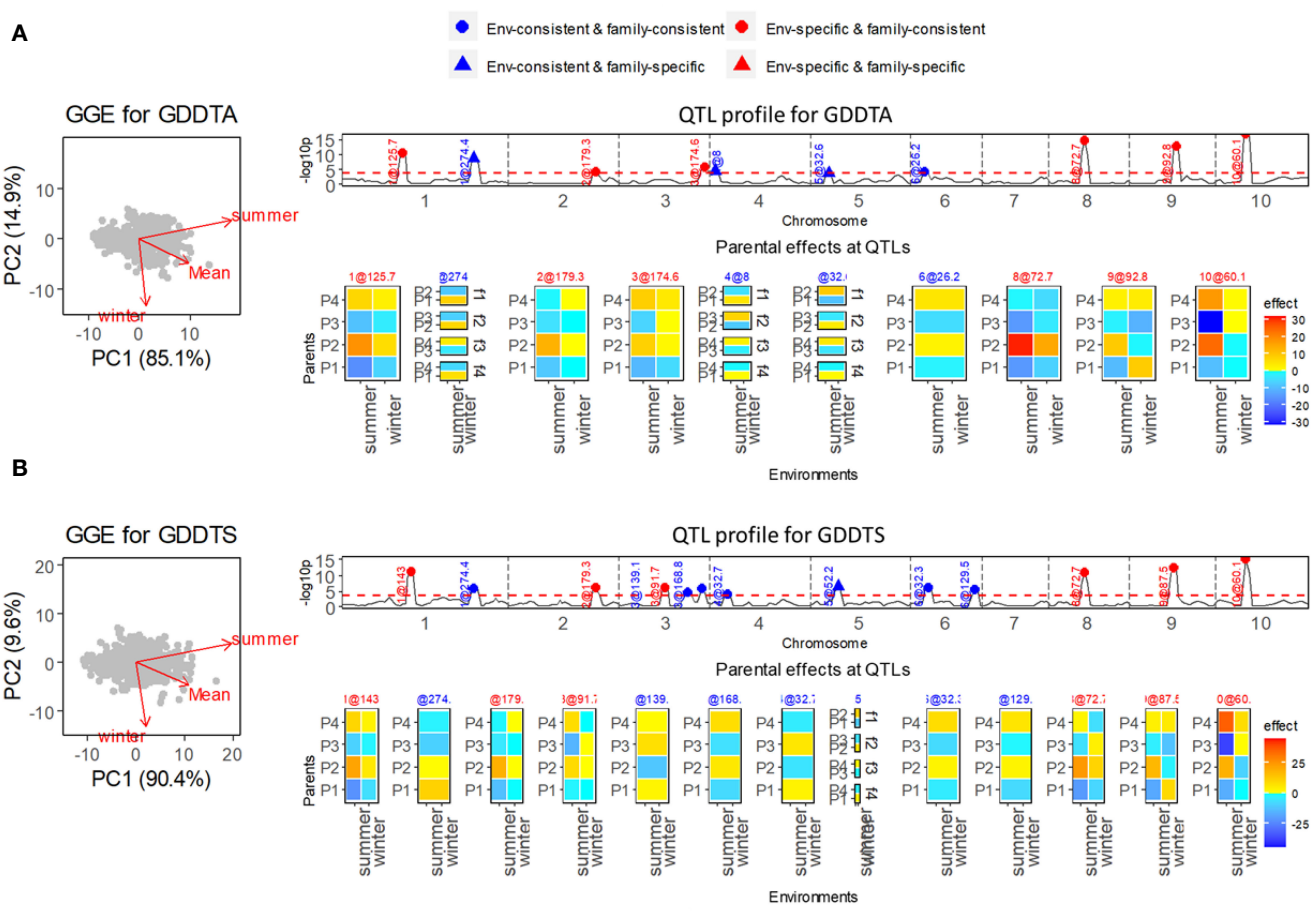
Results of QEI analysis of maize diallel. **(A)** Analysis for growing degree days to anthesis (GDDTA). **(B)** Analysis for growing degree days to silk (GDDTS). On the left, GGE biplot for exploring genetic correlations between environments. Top right shows the *−log10(p)* profile for QTL detection with superimposition of QTL allele effect types indicating consistency of allele effects across environments and families. Bottom right shows allele effect profiles with alleles being either consistent or inconsistent across environments and/or families. Environment-consistent QTLs show the same color and intensity across environments. Environment-specific QTLs show environment specific colors and/or intensities. Family consistent QTLs show a parent specific color and intensity that does not depend on the family and that is either consistent across environments or particular to each environment. Family-specific QTLs show colors and/or intensities for parents that change across families.

Two flowering-related traits, growing degree days to silking (GDDTS) and growing degree days to anthesis (GDDTA), were measured in the summer and winter seasons to evaluate the photoperiod sensitivity under long-day and short-day conditions. For both traits, the correlations between summer and winter conditions were weak (**Figures 2A, B**).

In the QEI analysis, we found that nearly half of the identified QTLs were environment-specific QTLs for both GDDTS and GDDTA. For the trait GDDTA (**Figure 2A**), in total 10 QTLs were detected, among which six QTLs were environment specific. Five of the six environment-specific QTLs were comparable with QTLs for photoperiodic responses (calculated as differences of responses between the two seasons) from a previous study (Coles et al., 2010). Particularly strong environment-specific QTLs on chromosomes 8, 9, and 10 were identified with contrasting parental effects in especially the summer season. The same summer season, QTLs were identified by Coles et al. (2010) using single-environment analysis. As for the trait GDDTS (**Figure 2B**), we identified six environment-specific QTLs out of a total of 13 QTLs, which were in accordance with the QTLs for photoperiodic responses identified by Coles et al. (2010) with strong effects under summer conditions.

For both traits GDDTA and GDDTS, a few environment-consistent QTLs were estimated with specific effects within families (EC&FS QTLs), which implied QTL-by-family background interactions. We did not identify ES&FS QTLs for GDDTA and GDDTS, but analysis of other traits, such as plant height (PH), ear height (EH), and total leaf number (TLN) in this maize diallel MET&MPP, revealed some ES&FS QTLs (results not shown here).

### 3.3 Maize NAM design (dent panel) for dry matter yield, days to silking, and plant height

**Figure 3:**
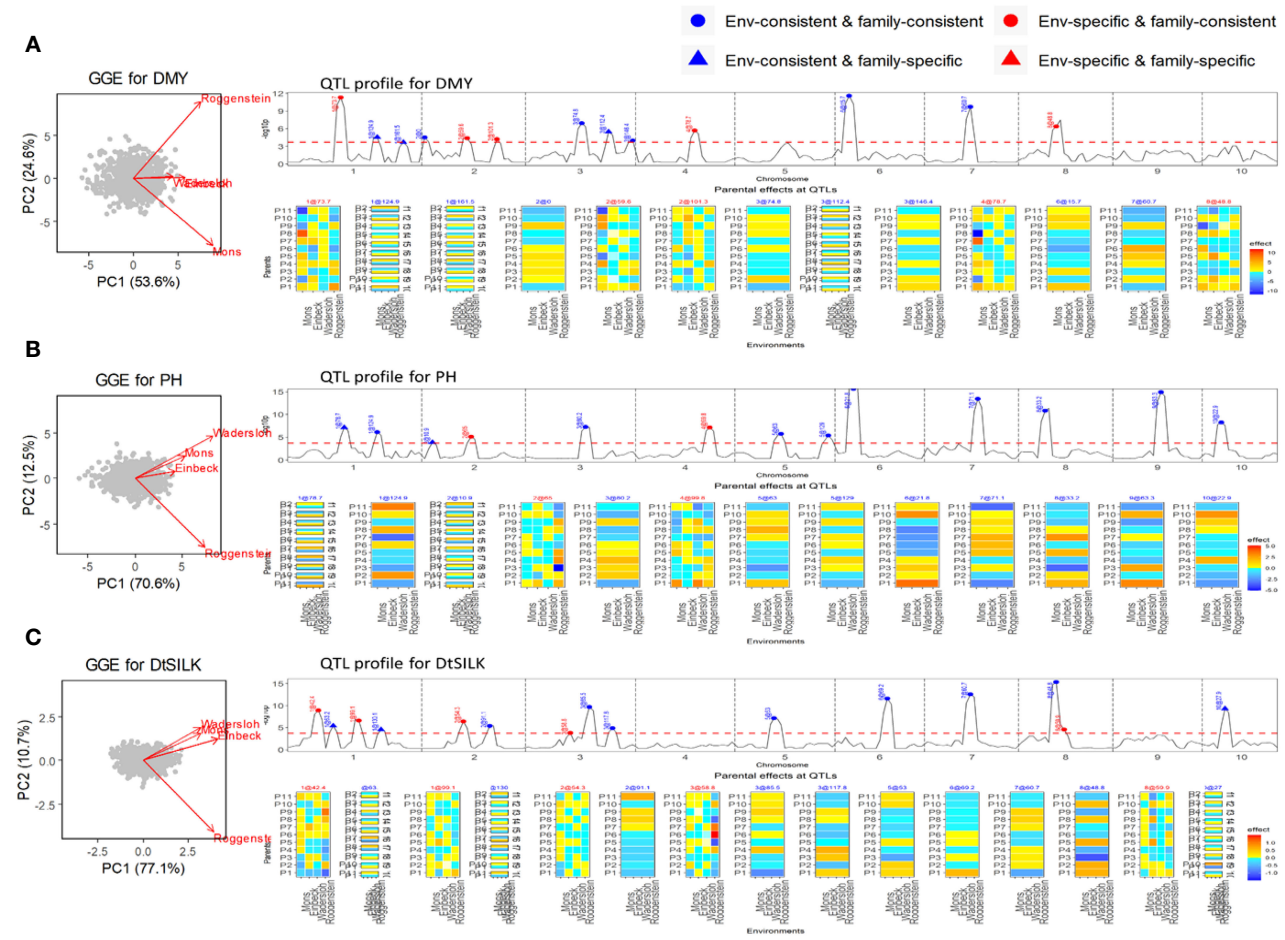
Results of QEI analysis of maize NAM dent design. **(A)** Dry matter yield (DMY), **(B)** Plant height (PH). **(C)** Days to silk (DtSILK). Interpretation of plots as in **Figure 2**.

The NAM dent panel of maize was screened for important traits including dry matter yield (DMY), plant height (PH), and days to silking (DtSILK) across four locations in the EU. The GGE plots (**Figure 3**) depict varying levels of genetic correlations between the environments. For DMY, the environments Roggenstein and Mons exhibited a weak correlation, whereas the environments Einbeck and Wadersloh displayed a highly positive correlation. For PH, the Roggenstein environment stood apart with a weak correlation to the remaining environments. Regarding DtSILK, the environments Wadersloh, Mons, and Einbeck formed a cluster with a weak correlation to Roggenstein.

In our QEI analysis, we successfully identified several environment-specific QTLs for each trait. For instance, our methodology detected five environment-specific QTLs out of a total of 13 QTLs for the DMY trait (**Figure 3A**). The effect profiles of these five environment-specific QTLs exhibited strong variations between the two weakly correlated environments, Mons and Roggenstein. In a separate study, Giraud et al. (2014) conducted combined linkage disequilibrium linkage analysis (LDLA) using adjusted means across all environments. The environment-consistent QTLs identified through our QEI analysis were comparable with those QTLs on chromosome 1, 3, 6, and 7 reported by Giraud et al. (2014). However, it is worth noting that our study revealed environment-specific QTLs on chromosomes 1, 2, and 4 that were not previously reported by Giraud et al. (2014). For the PH trait, we found two environment-specific QTLs out of a total of 13 QTLs (**Figure 3B**), and these QTLs displayed differential effect profiles between the two weakly correlated environments, Roggenstein and Wadersloh. Regarding the DtSILK trait, our analysis identified five environment-specific QTLs out of a total of 16 QTLs (**Figure 3C**), which exhibited distinct effect profiles between Roggenstein and the other environments.

For all traits, we detected QTLs with consistent effects across environments that could be either consistent across families as well as specific to families (EC&FC and EC&FS QTLs), but no environment-specific and family-specific (ES&FS) QTLs were found for any of the traits.

### 3.4 Maize NAM design (flint panel) for dry matter yield, days to silking and plant height

**Figure 4:**
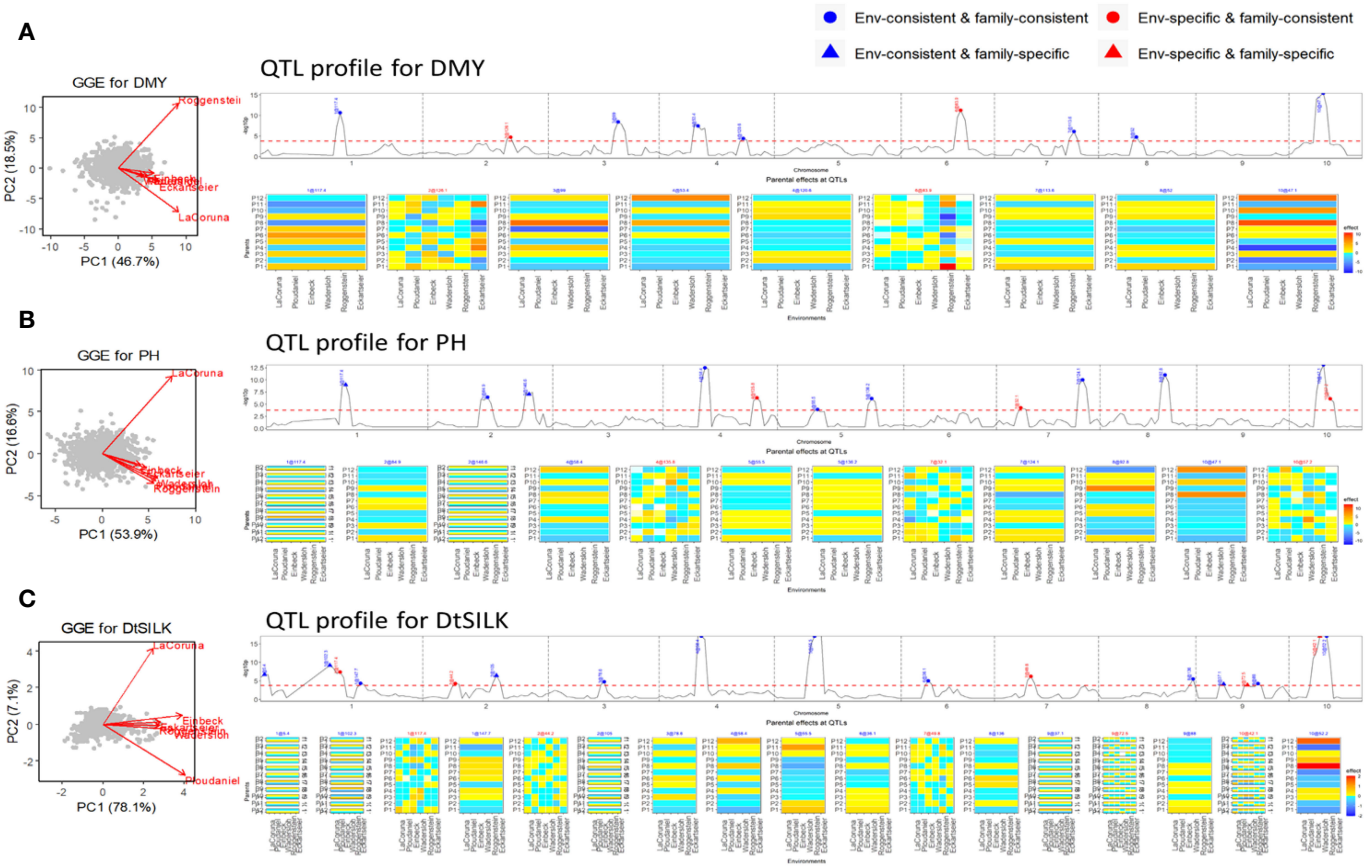
Results of QEI analysis of maize NAM flint design. **(A)** dry matter yield (DMY), **(B)** plant height (PH), and **(C)** days to silk (DtSILK). Interpretation of plots as in **Figure 2**.

The flint panel of the maize NAM design was screened for DMY, PH, and DtSILK across six locations in the EU. For the DMY trait (**Figure 4A**), the GGE biplot reveals three clusters: La Coruña, Roggenstein, and the remaining environments. Among the detected nine QTLs, two QTLs on chromosomes 2 and 6 were found to be specific to certain environments. These environment-specific QTLs were previously studied by Garin et al. (2020), who assessed QEI between the weakly correlated environments La Coruña and Roggenstein. Our results corroborate Garin’s work by confirming an environment-specific QTL on chromosome 6 with significant effects in the Roggenstein environment. The QEI signal identified by Garin et al. (2020) on chromosome 5, displaying a relatively weak signal, was not detected in our analysis across the six environments. We discovered an additional environment-specific QTL on chromosome 2, which exhibited different effect profiles between Eckartseier and the other environments. This particular QTL was not reported in the study by Garin et al. (2020), which only considered the locations of Roggenstein and La Coruña.

For PH (**Figure 4B**), two QTLs were family-specific while being environment-consistent (EC&FS). For DtSILK (**Figure 4C**), we even found four such EC&FS QTLs. Furthermore, for this trait, we also identified two QTLs that were both environment- and family-specific (ES&FS).

### 3.5 NIAB diverse wheat MAGIC

**Figure 5:**
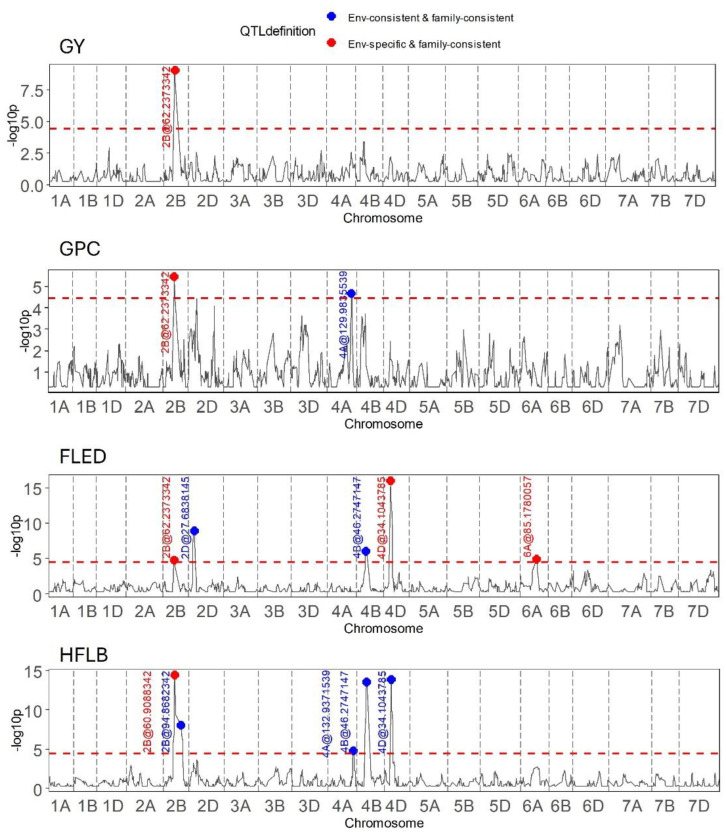
Results of QEI analysis in wheat MAGIC for the traits grain yield (GY), grain protein content (GPC), flag leaf to ear distance (FLED), and height to flag leaf base (HFLB).

Outcomes of genome wide QTL scans for two grain traits (GY, GPC) and two plant height related traits (HFLB, FLED) are presented in **Figure 5**. The most remarkable result of these scans is that on chromosome 2B, around 62 Mb, QEI was detected for all four traits with the direction of the allelic effect tending to reverse for the majority of founder alleles between the two seasons (**Table 2**). Notably, founder Kloka showed the most extreme QEI at this locus for all four traits. With respect to the two grain-related traits, the Kloka allele showed a strong negative effect for GY in 2016–2017, and a light negative effect in 2017–2018, whereas for GPC, the Kloka allele effect was strongly positive in 2016–2017 and lightly positive in 2017–2018. Therefore, a strong negative correlation could be observed between the Kloka allele effects for GY and GPC at this locus, as would be expected from well-established trade-offs between yield and protein content in wheat (e.g., Scott et al., 2021; White et al., 2022). For the plant height traits, the QTL effect from Kloka at this locus on chromosome 2B reduced HLFB across both seasons, but very strongly in 2016–2017 and somewhat in 2017–2018. For FLED, the Kloka allele effect was moderately positive in 2016–2017 and somewhat negative in 2017–2018. Previous studies show that the long-day photoperiod response locus *Photoperiod-B1* (*Ppd-B1*) is located at this approximate genome position and that it affects flowering time in the NIAB Diverse MAGIC population (Scott et al., 2021). Allelic effects at *Ppd-B1* are known to be controlled by copy number variation (CNV) in the underlying gene, whereby wild-type photoperiod-sensitive *Ppd-B1b* alleles are associated with a haploid CNV of 1, whereas photoperiod-sensitive *Ppd-B1a* alleles that result in earlier flowering are associated with elevated copy number variation (e.g., CNV = 2 in the cultivar Récital, CNV = 3 in Sonora64, and CN0V = 4 in Chinese Spring) (Díaz et al., 2012). To further determine whether allelic variation at *Ppd-B1* may underpin the environment-specific interactions at this genomic region for GY, GPD, HFLB, and FLED, we assessed *Ppd-B1* CNV in the 16 NIAB Diverse MAGIC founders. Using quantitative TaqMan^®^ assay, we found that whereas 14 founders carried one copy (indicative of wild-type *Ppd-B1b* alleles), two founders had increased CNV indicative of photoperiod insensitive *Ppd-B1a* alleles associated with early flowering: Kloka (CNV = 3) and Maris Fundin (CNV = 4).

**Table 2:** Heat plot for parental allele effects estimated for four traits in wheat MAGIC in two environments (2016–2017, 2017–2018) at chromosome 2B close to *PpdB1* locus.

Season	Trait							
	GY		GPC		HFLB		FLED	
	2016–2017	2017–2018	2016–2017	2017–2018	2016–2017	2017–2018	2016–2017	2017–2018
Parent								
Banco	−0.03	0.06	0.01	−0.07	−0.18	−0.46	0.08	−0.10
Bersee	−0.18	0.01	−0.02	0.00	0.48	−0.03	−0.08	0.19
Brigadier	0.14	−0.06	0.00	0.10	0.75	1.29	0.23	0.02
Copain	0.10	0.01	−0.02	−0.05	1.54	0.57	−0.33	0.20
Cordiale	0.12	0.00	−0.15	0.02	−0.47	−0.48	0.17	−0.25
Flamingo	−0.03	−0.03	0.19	0.00	0.54	−0.47	−0.08	0.05
Gladiator	0.00	0.02	0.04	−0.04	−0.06	0.53	−0.16	0.00
Holdfast	0.02	−0.01	0.24	−0.02	−0.90	0.85	0.20	−0.12
Kloka	−0.44	−0.02	0.35	0.09	−4.11	−1.51	0.64	−0.22
Maris Fundin	−0.01	0.00	0.01	−0.02	−0.47	−1.30	−0.27	0.01
Robigus	0.01	0.03	−0.01	−0.04	0.32	−0.12	−0.40	0.10
Slejpner	−0.01	−0.02	−0.12	0.06	−0.03	0.55	0.21	0.05
Soissons	0.10	0.00	−0.23	−0.01	0.63	0.58	−0.12	0.12
Spark	0.12	−0.06	−0.11	0.13	0.30	−0.27	0.38	−0.28
Steadfast	0.04	0.03	−0.09	−0.09	0.93	−0.08	−0.31	−0.02
Stetson	0.03	0.04	−0.09	−0.06	0.74	0.35	−0.16	0.27

The color scales are defined per trait to run from strongly negative (deep blue), to moderately negative (light blue), neutral (white), moderately positive (light red), to strongly positive (deep red).

## 4 Discussion

### 4.1 QEI analysis by assessing the stability of QTL effects across families and environments

The MET&MPP analysis employs IBD-based mixed model approaches to detect QEI by testing various types of QTL effects across diverse environments and families. Previous studies have discussed the advantages of utilizing multiallelic IBD markers compared with biallelic identity-by-state (IBS) markers (Jurcic et al., 2021; Li et al., 2021), as well as the distinction between modeling QTL terms as random or fixed effects (Boer et al., 2007; Wang et al., 2022). In our current study, we propose fitting functions of IBD probabilities as genetic predictors within the MET&MPP analysis. This approach enables the estimation of multiallelic QTL effects with respect to parent origins, considering the expected numbers of allele copies from each parent (Wei and Xu, 2016; Li et al., 2021). In contrast to modeling fixed QTL effects in the MET&MPP analysis, treating QTL effects as random terms addresses the issue of overfitting the number of genetic parameters, particularly in complex MET&MPPs involving a large number of parents, families, and environments. Our QTL modeling approach allows for defining the nature of random QTL effects, such as EC&FC, ES&FC, EC&FS, and ES&FS, by modeling combinations of homogeneity or heterogeneity of genetic structures corresponding to both environments and families.

EC&FC QTLs can be regarded as the most stable QTLs in the MET&MPP analysis. EC&FC QTLs are comparable with those QTLs identified by an analysis on genotypic means across environments (Giraud et al., 2014; Garin et al., 2020). EC&FC QTLs identified in MET&MPP analyses at particular genomic positions will in general agree with QTLs for the same trait identified in single-environment analyses, like those in Coles et al. (2010), but there is no guarantee that all the QTLs identified in simpler types of analyses will be reproduced by a more complex MET&MPP analysis. EC&FC QTLs tend to be detected with higher power and resolution in MET&MPP analysis than when analyzing separate environments or a genotypic main effect across environments, as the joint analysis of all environments increases the sample size for mapping stable QTLs.

QTLs detected in separate environments with differential mapping profiles are likely to be detected as ES&FC QTLs by MET&MPP analysis. Parental effect profiles of ES&FC QTLs across environments tend to show differences between weakly correlated environments, whereas highly and positively correlated environments rarely convey QEI signals. Positions for ES&FC QTLs in our MET&MPP analyses coincided with QTLs found by a simpler single trait/environment QTL procedure in which the response variable was the difference between two environments, such as in the maize diallel MPP (Coles et al., 2010). However, we remark that with small population sizes, weak QEI signals that occur in a limited number of environments may not be detected as these signals are diluted by the noise in all those environments in which the QTL did not show an effect. For example, in the maize NAM flint panel (811 genotypes across 6 environments), the QEI signal on chromosome 5 specific to the environments La Coruña and Roggenstein (Garin et al., 2020) disappeared in our MET&MPP analysis on the full set of six environments, but this QTL was indeed identified when the MET&MPP analysis was restricted to La Coruña and Roggenstein.

Defining QTL effects to vary across families allows the evaluation of QTL-by-family interactions (QFI) (Jannink and Jansen, 2001; Blanc et al., 2006; Han et al., 2016). For family-specific QTLs that are stable across environments (EC&FS), simple digenic interaction can be investigated for pairs of markers by two-dimensional genome scans in families. Such two-dimensional QTL scans for epistatic interactions require large family sizes for sufficient detection power. For the detection of ES&FS QTLs with family-specific effects that are unstable across environments, even larger families are required. As an illustration of this point, we detected very few ES&FS QTLs in the maize diallel design, with around 140 genotypes per family over two environments, and hardly any ES&FS QTL in the maize NAM design, with around 80 genotypes per family.

### 4.2 Pleiotropic effects of QTL at *Ppd-B1* in the NIAB Diverse MAGIC population

In a previous paper (Scott et al., 2021), it was shown that the photoperiod response locus *Ppd-B1* affects the time taken to reach key developmental stages in the NIAB Diverse MAGIC population, including flag leaf emergence (growth stage 39, GS39), ear emergence (GS55), and flowering time (GS65). Under field conditions, the increasing day length across spring and summer help trigger transition from vegetative to reproductive plant growth and rate of progress across all subsequent developmental stages. Thus, allelic variation at *Ppd-B1* helps define the timepoints that key development stages are exposed to the prevailing environmental conditions. Here, we identified QEI at a pleiotropic QTL controlling multiple plant height and grain traits at the *Ppd-B1* locus, finding QTL effects from the founder Kloka to be both notably strong, and affected by environment. Kloka was unique among the NIAB Diverse MAGIC founders in that it carried three copies of the *Ppd-B1* gene, predictive of an early flowering photoperiod insensitive *Ppd-B1a* allele. Interestingly, no QTL for total plant height was identified in the population at *Ppd-B1* (Scott et al., 2021). Together, these results indicate that *Ppd-B1a* alleles promote flowering and that whereas overall height is not affected, under some growth environments, the Kloka photoperiod-insensitive *Ppd-B1a* allele results in a shift in plant height ratios between the upper and lower stems and is associated with effects on final grain yield and protein content. In environment 1, height to flag leaf base (HFLB) showed a strong decrease whereas height from flag leaf to ear base (FLEB) exhibited a marked increase, whereas in environment 2 the effects of the Kloka *Ppd-B1a* allele were much reduced. While the two field trials investigated here were located on the same farm in the UK, analysis of weather data has shown that both years were unusual compared with historical data across the preceding 56 years: environment 1 was characterized by extreme high temperatures and drought in the developmental stages before anthesis (March and April), and extreme precipitation during grain filling, whereas environment 2 was characterized by extreme terminal heat and drought during the grain filling stage (June and July) (Fradgley et al., 2023). Thus, the combined effects of the early flowering *Kloka Ppd-B1a* allele in environment 1 would have led to earlier development of the reproductive meristem, stem extension, and mid-canopy emergence during extreme temperatures and low precipitation. As temperature was unusually high throughout anthesis and start of grain filling (May–June) in both test environments, and subsequent grain filling stages in environment 1 took place under average temperatures, it is not likely that the known negative impact of high temperatures around anthesis and grain filling on wheat yield (e.g., Djanaguiraman et al., 2020) are the cause of reduced yield and high grain protein content in environment 1. Interestingly, analysis of CNV at *Ppd-B1* genes in tetraploid wheat show that the photoperiod-insensitive *Ppd-B1b* allele results in increased spikelet number per ear (Arjona et al., 2018). This suggests that the pronounced negative effect of allelic variation at *Ppd-B1* on yield in environment 1 may have been due to earlier exposure of each developmental stage to the extreme heat and drought conditions across the stem extension phases across which final spikelet number is determined in the shoot apical meristem as it reaches the terminal spikelet phase (as well as possible impacts of changed stem length ratios on later development during grain filling).

In addition to Kloka, the only other founder to have increased *Ppd-B1* CNV was Maris Fundin, predicted to carry four copies via TaqMan^®^. While increased CNV is associated with early flowering photoperiod-insensitive *Ppd-B1a* alleles in wheat (Díaz et al., 2012; Würschum et al., 2015), other factors may influence the allelic effect. For instance, previous characterization of the photoperiod-insensitive *Ppd-B1a* allele in Chinese Spring found that it carries four tandemly duplicated copies of the gene: three intact and one truncated (Díaz et al., 2012). Indeed, analysis of three sets of near isogenic lines (NILs) in which photosensitive *Ppd-B1a* alleles from either Chinese Spring (CNV = 4), Sonora64 (CNV = 3), or Récital (CNV = 2) had been introgressed into a Paragon genetic background found the insensitive allele from Sonora64 to be significantly earlier flowering than that from Chinese Spring (Díaz et al., 2012). This highlights that whereas increased copy number results in an early flowering allele, the allelic effect is not wholly dependent on the number of copies of the genes present and that functionality of the copies present also plays a role. Indeed, whereas we found the NIAB Diverse MAGIC founder Maris Fundin to have four copies of *Ppd-B1*, analysis of allelic effects found that it did not have as strong an effect on the four target grain and plant height traits compared with the Kloka allele (CNV = 3). Surveys of >1,100 global wheat varieties (Díaz et al., 2012; Kane et al., 2013; Würschum et al., 2015) finds that the occurrence of cultivars with four copies of *Ppd-B1* is very rare, having previously been identified in only a limited number of Australian cultivars with pedigree links to Chinese Spring (Kane et al., 2013). Comparison of the pedigree of Maris Fundin (Capelle Desprez × [Vilmorin-29 × Vogel-8058]) × TJB-16 (Fradgley et al., 2019), with cultivars previously assayed for *Ppd-B1* CNV, did not enable insight into the parental donor of the Maris Fundin CNV. However, collectively our results indicate that while the Maris Fundin allele characterized by the presence of four *Ppd-D1* copies likely confers a photoperiod-insensitive *Ppd-B1a* allele, its effect is not as strong as the CNV = 3 Kloka allele identified here.

### 4.3 Conclusion

We introduced a general framework for studying QEI in METs for MPPs. The framework creates design matrices for QTL effects starting from IBD probabilities between parents and offspring for any kind of MPP. The IBD probabilities are combined with a standard procedure for creating factorial interactions between quantitative (IBD for genomic position) and qualitative (environment, family) covariates. In this way, four types of design matrices are produced that correspond to four types of QTL effects: EC&FC, ES&FC, EC&FS, and ES&FS. The effects are assumed to come from normal distributions. Following up on the definition of the QTL models, a relatively simple stepwise protocol is followed to arrive at multi-QTL models with mixtures of QTL effect types at the different loci. We illustrated with various examples the power of our approach to dissect the genetic basis of GEI in any kind of MPP.

## Data availability statement

Publicly available datasets were analyzed in this study. This data can be found here: https://git.wur.nl/li178/QxE_MPP.

## Author contributions

WL: Conceptualization, Data curation, Formal analysis, Investigation, Methodology, Software, Validation, Visualization, Writing – original draft. MB: Conceptualization, Formal analysis, Investigation, Methodology, Software, Supervision, Validation, Visualization, Writing – original draft, Writing – review & editing. RJ: Conceptualization, Funding acquisition, Resources, Supervision, Writing – review & editing. CZ: Conceptualization, Formal analysis, Investigation, Methodology, Software, Supervision, Writing – review & editing. LPA: Data curation, Investigation, Validation, Writing – original draft. JC: Data curation, Investigation, Validation, Writing – original draft. FV: Conceptualization, Formal analysis, Funding acquisition, Investigation, Methodology, Project administration, Resources, Supervision, Validation, Writing – original draft, Writing – review & editing.
